# Formulation of polylactide-co-glycolic acid nanospheres for encapsulation and sustained release of poly(ethylene imine)-poly(ethylene glycol) copolymers complexed to oligonucleotides

**DOI:** 10.1186/1477-3155-7-1

**Published:** 2009-04-07

**Authors:** Shashank R Sirsi, Rebecca C Schray, Margaret A Wheatley, Gordon J Lutz

**Affiliations:** 1 Drexel University College of Medicine, Department of Pharmacology and Physiology, Philadelphia, Pennsylvania 19102, USA; 2 Drexel University, School of Biomedical Engineering, Philadelphia, Pennsylvania 19104, USA

## Abstract

Antisense oligonucleotides (AOs) have been shown to induce dystrophin expression in muscles cells of patients with Duchenne Muscular Dystrophy (DMD) and in the *mdx *mouse, the murine model of DMD. However, ineffective delivery of AOs limits their therapeutic potential. Copolymers of cationic poly(ethylene imine) (PEI) and non-ionic poly(ethylene glycol) (PEG) form stable nanoparticles when complexed with AOs, but the positive surface charge on the resultant PEG-PEI-AO nanoparticles limits their biodistribution. We adapted a modified double emulsion procedure for encapsulating PEG-PEI-AO polyplexes into degradable polylactide-co-glycolic acid (PLGA) nanospheres. Formulation parameters were varied including PLGA molecular weight, ester end-capping, and sonication energy/volume. Our results showed successful encapsulation of PEG-PEI-AO within PLGA nanospheres with average diameters ranging from 215 to 240 nm. Encapsulation efficiency ranged from 60 to 100%, and zeta potential measurements confirmed shielding of the PEG-PEI-AO cationic charge. Kinetic measurements of 17 kDa PLGA showed a rapid burst release of about 20% of the PEG-PEI-AO, followed by sustained release of up to 65% over three weeks. To evaluate functionality, PEG-PEI-AO polyplexes were loaded into PLGA nanospheres using an AO that is known to induce dystrophin expression in dystrophic *mdx *mice. Intramuscular injections of this compound into *mdx *mice resulted in over 300 dystrophin-positive muscle fibers distributed throughout the muscle cross-sections, approximately 3.4 times greater than for injections of AO alone. We conclude that PLGA nanospheres are effective compounds for the sustained release of PEG-PEI-AO polyplexes in skeletal muscle and concomitant expression of dystrophin, and may have translational potential in treating DMD.

## Introduction

Steric block antisense oligonucleotides (AOs) are considered potential therapeutics for a variety of diseases due to their capacity to modulate alternative splicing, correct aberrant mRNA splicing, and induce exon skipping [1-5]. Duchenne muscular dystrophy (DMD), a fatal disease caused by mutations in the gene encoding dystrophin, has been established as an excellent candidate for AO-based treatment [6-9]. The main barrier that has limited the usefulness of AOs in treatment of DMD, and most other diseases, is the inability to deliver them to their target cell nuclei.

Poly(ethylene imine) (PEI) is a highly protonatable amine rich polymer that has been established as an efficient nucleotide carrier [10]. The cationic nature of PEI allows the polymer to interact with both the negatively-charged phosphate backbone of nucleotides and the negatively-charged elements of cell membranes, promoting endocytotic uptake of the nucleotides into cells [10-17]. Grafting of polyethylene glycol (PEG) polymers to PEI has been shown to significantly enhance its functionality as a nucleotide carrier by reducing cytotoxicity and improving biocompatibility [13-15,18]. Overall, PEG-PEI copolymers represent an adaptable nucleotide delivery system with controllable size and surface charge, and flexibility for addition of moieties that target specific entities on cell membranes.

Cationic PEI-based transfection agents have repeatedly been demonstrated to be one of the most effective non-viral vectors for facilitating uptake of nucleic acid based compounds *in vitro *due to a so called "proton sponge" effect [10,16,19-21]. However, although PEG-PEI copolymers have been demonstrated to facilitate delivery of AOs *in vivo*, [22-25], the cationic surface charge of polyplexes which enables cellular uptake, likely limits their biodistribution by non-specific binding to components in the blood and extracellular environment [26,27]. Methods of shielding the cationic surface charge of polyplexes, followed by sustained release, could overcome these limitations.

Biodegradable poly(lactic-co-glycolic acid) (PLGA) polymers are versatile and biocompatible compounds that have been FDA approved and utilized in a wide variety of drug delivery applications including the encapsulation of nucleic acids [28-30]. The properties of PLGA nanospheres can be controlled by utilizing a range of PLGA chemistries and altering the nanosphere synthesis conditions, producing nanospheres with variable release kinetics and sizes [28,30-32]. Nanosized PLGA has been formulated for encapsulation of naked plasmid DNA and AO [33,34]. Also, PEI has been successfully encapsulated into PLGA nanospheres for intranasal delivery of genes to pulmonary epithelial cells [35], and oral delivery of oligonucletides as an immunostimulant [36], or simply to improve *in vitro *transfection efficiency [37,38]. Previously, cationic polymers (PEI or polylysine) complexed with nucleic acids have been encapsulated into PLGA, however most of these compounds were restricted to micron sized spheres [39-44]. For many drug delivery applications however, it would be preferential to encapsulate PEI within nanosized spheres. Nanoparticles may be preferred over microparticles as delivery vehicles due to more favorable circulation times as well as biodistribution. Small nanoparticles which can be internalized within cells are also useful for cytosolic delivery of compounds that cannot readily cross cell membranes, such as nucleic acids [45]. Recently, one study has demonstrated the encapsulation of PEI within PLGA nanospheres, showing some improvement in transfection efficiency and cell viability, however, this study is limited to cultured cells [46].

Presently we studied formulation parameters for the encapsulation of PEG-PEI-AO polyplexes within PLGA nanospheres, and demonstrated their functionality *in vivo*. PEG-PEI-AO polyplexes were encapsulated within PLGA nanospheres, effectively shielding the cationic surface charge of the polyplexes, and permitting their sustained release. Injections of PEG-PEI-AO encapsulated into PLGA nanospheres in limb musculature of *mdx *mice resulted in improvement in number of dystrophin-positive fibers compared to AO alone. The results of this proof-of-concept study demonstrate the feasibility of encapsulating PEG-PEI-AO polyplexes within PLGA nanospheres, which may potentially be used for sustained release of the polyplexes over time or improving the efficiency of systemic polyplex delivery. These compounds represent promising agents for delivery of AO to dystrophic skeletal muscle and may find usage in treatment of DMD.

## Methods

### Synthesis of PEG-PEI copolymers

Details of the synthesis of the PEG-PEI copolymers, as well their physicochemical properties when complexed with AO were previously described [47]. Briefly, copolymers composed of branched poly(ethylene imine)-25000 (PEI25K) and methoxypoly(ethylene glycol)-5000 (mPEG5K) (Sigma-Aldrich, St. Louis, MO, USA) were prepared using a two-step procedure [13]. First, mPEG5K was activated with hexane-1,6-diisocyanate. Second, PEI25K and activated mPEG5K were reacted at a PEG:PEI molar ratio of 10:1. Throughout the manuscript we refer to this PEI25K(PEG5K)_10_copolymer simply as PEG-PEI.

### Synthesis of PLGA nanospheres containing PEG-PEI-AO polyplexes

Encapsulation of PEG-PEI-AO polyplexes into PLGA nanospheres was carried out using a double emulsification (water-in-oil-in water) technique [48]. PLGA polymers of 72, 50, and 17 kDa were used (all 50:50; Lakeshore Biomaterials, Birmingham, AL, USA). The 72 kDa PLGA was lauryl ester end-capped, while the other MWs were not. PLGA (70 mg) was dissolved in 2 mL chloroform (Sigma Aldrich) to form the organic phase, and then sonicated on ice for 30 seconds at 51 Watts with a microtip attachment (Ultrasonics W-385 Sonicator; Heat Systems, Farmingdale, NY, USA). PEG-PEI-AO polyplexes were prepared by mixing PEG-PEI with a 2'O-methyl AO ('5-GGCCAAACCUCGGCUUACCU-3'; Trilink Biotechnologies, San Diego, CA) at a nitrogen to phosphate (N:P) ratio of 5:1, as previously described [47]. The primary aqueous phase contained either PEG-PEI-AO (1 mg of AO), AO alone (1 mg), or only distilled and deionized water (DI H_2_O) in a volume of 300 μl. The primary aqueous phase was then added to the organic phase, and emulsified by sonication for 30 seconds at 30–52 Watts on ice. The resultant water-in-oil emulsification was added dropwise into a 25 mL solution of cold (4°C) 5% polyvinyl alcohol (22 kDa, 88% Hydrolyzed; Acros Chemicals; Morris Plains NJ) in a 50 mL glass beaker while stirring at 400 rpm to form the secondary emulsion. In the initial samples, the secondary emulsion was sonicated for 1 minute on ice at either 38 W or 52 W. Nanospheres with a smaller mean size and more uniform size distribution were subsequently obtained by splitting the secondary emulsion into equal volumes in three 20 mL glass scintillation vials, sonicating each for 1 minute at 52 W on ice, and recombining the solutions. Chloroform was removed by evaporation overnight at room temperature while stirring at 400 rpm. The resultant nanoparticles were collected by high speed ultracentrifugation (Ultra 80 Ultracentrifuge; Sorvall, Asheville, NC, USA) at 20000 rpm using an AH-627 rotor (Sorvall) and appropriate buckets (53,300 g). Particles were washed twice with DI H_2_O and collected. The nanospheres were then resuspended in DI H_2_O, and lyophilized for 72 hours (Virtis Gardiner, NY, USA) prior to storage at -20°C.

### Particle size and surface charge

PLGA nanospheres were suspended in a diluted PBS solution (1× PBS diluted 1:800 in DI H_2_O, adjusted to pH 7.2). The nanospheres were suspended at a concentration of 0.1 mg/ml in the diluted PBS solution. Particle size and surface charge were measured by dynamic light scattering (DLS) and zeta potential, respectively (Zetasizer; Malvern Instruments, Southborough, MA, USA). All measurements were made in triplicate.

### Encapsulation efficiency and release kinetics

The amount of AO encapsulated within PLGA nanospheres (encapsulation efficiency; EE) was determined by spectrophotometry. PLGA nanospheres encapsulated with PEG-PEI-AO (1 mg AO) were dissolved in 500 μL of 0.5 M NaOH to release the encapsulant, centrifuged for 30 minutes at 16,000 g (5415D; Eppindorf, Westbury, NY. USA), and the absorbance of the supernatant was measured at 260 nm using a low volume quartz cuvette (Ultraspec 2100; Amersham Biosciences, Piscataway, NJ, USA). The concentration of AO in the PLGA samples was determined by comparison with a standard curve generated from AO at varying concentrations dissolved in 0.5 M NaOH.

To determine release kinetics, PLGA nanospheres encapsulated with PEG-PEI-AO were suspended in sterile PBS (1 mg/ml; pH 7.2) and incubated while rotating at 37°C. At the desired time points, samples were centrifuged for 30 minutes at 16 g, the supernatant was discarded, and the pellet was dissolved in 0.5 M NaOH. AO concentration was measured as described for EE. Both EE and release kinetics measurements were made in triplicate.

### Intramuscular injections of mdx mice

All experiments were performed on male *mdx *mice 6–8 wks of age (C57BL/10ScSn-Dmdmdx/J) or age-matched normal male mice (C57BL/10SnJ) (Jackson Laboratories, Bar Harbor, ME, USA). Mice were anesthetized with ketamine/xylazine and monitored according to approved NIH and university guidelines. Tibialis anterior (TA) muscles (N = 4 per group) were injected with either PLGA-PEG-PEI-AO, PEG-PEI-AO or AO alone dissolved in 15 μl of sterile saline as previously described [24,25]. For all groups the AO dose was 5 μg per injection. After recovery from anesthesia, mice were returned to normal cage activity. All treated mice received injections on day 0, 3, and 6 and TA muscles were harvested 3 weeks after the initial injection. Muscles were isolated, pinned to parafilm-covered cork, snap frozen in liquid N_2_-cooled 2-methylbutane, and stored at -80°C until further processing. Control muscles were harvested from uninjected age-matched *mdx *and normal mice.

### Immunohistochemistry and histology

Transverse frozen sections (10 μm) were obtained from TA muscles using a cryostat (Leica CM 3050 S, Bannockburn, IL, USA). Dystrophin immunolabeling was performed on frozen sections using a rabbit polyclonal anti-dystrophin antibody, (1:125; Abcam Inc., Cambridge, MA, USA), which labels the C-terminus of dystrophin. The secondary antibody was Cy3-Anti-Rabbit IgG (1:500; Jackson Immuno Research). Immunosections were counterstained with Hoechst dye (Sigma) to visualize nuclei. Routine hemotoxylin and eosin (H & E) staining was used to examine overall muscle morphology and assess the level of infiltrating mononucleated cells.

Dystrophin-immunolabeled transverse sections obtained from the midpoint along the length of TA muscles were imaged as whole sections using a color imaging camera (SPOT RT; Diagnostic Instruments, Sterling Heights, MI, USA) mounted on an MZFL3 stereomicroscope (Leica). The number of dystrophin-positive fibers in entire muscle cross-sections was counted using the cell counter function of ImageJ software .

### Statistical Analysis

All data are reported as mean values ± SEM. Statistical differences between treatment groups were evaluated by ANOVA (Statview; SAS Institute, Cary, NC).

## Results and discussion

**Figure 1:**
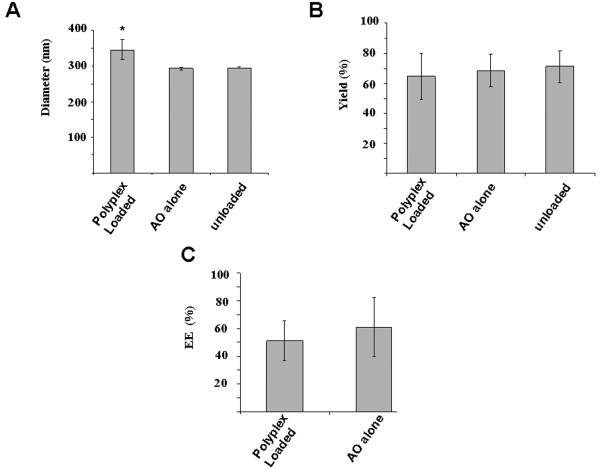
**Characterization of 72 kDa lauryl ester end-capped PLGA nanospheres with and without encapsulated PEG-PEI-AO polyplexes or AO alone**. (**A**) Mean diameter determined by DLS. PLGA nanospheres loaded with PEG-PEI-AO polyplex were significantly larger than those loaded with AO alone or unloaded samples (*P = .0079). (**B**) Polymer yields calculated based on the weight of the resultant nanospheres and initial mass of PLGA and encapsulant. (**C**) Encapsulation efficiency (EE). Three separate samples were evaluated for each group.

The goal of this study was to evaluate the feasibility of encapsulating PEG-PEI-AO polyplexes within biodegradable PLGA nanospheres for the purpose of improving delivery of these cationic polyplexes *in vivo*. We initially used a double emulsion procedure as outlined by Cohen-Sacks et. al., [48] to encapsulate either PEG-PEI-AO, AO alone, or water (unloaded) into 72 kDa ester end-capped PLGA. DLS measurements showed both AO-loaded and unloaded PLGA nanospheres had nearly the same size (292.5 ± 3.1 and 294.9 ± 2.4 nm, respectively; Figure 1A), and were similar in size to previously reported nanospheres loaded with naked phosphorothioated AO [48]. However, nanospheres loaded with PEG-PEI-AO polyplexes showed significantly higher mean diameters (345.4 ± 28.4 nm) compared to unloaded nanospheres (P < 0.01). This result is consistent with De Rosa et al [39,49] who observed a marked size increase in micron-sized PLGA when PEI-AO polyplexes were introduced into the primary aqueous phase of the emulsion. The average yield for PLGA nanospheres loaded with PEG-PEI-AO, AO, or unloaded was 71.0 ± 10.5%, 68.5 ± 10.8%, and 64.5 ± 15.10%, respectively, with no significant difference between groups (P > 0.05; Figure 1B). The EE for polyplex-loaded and AO-loaded nanospheres was 51.3 ± 14.4% and 60.3 ± 21.3%, respectively (Figure 1C).

Additionally, larger aggregates were apparent in samples containing PEG-PEI-AO that were not seen in samples prepared without PEG-PEI-AO (data not shown). While these particles were easily filtered out with a 1 μm syringe filter, the size distribution of the filtered sample remained remarkably non-uniform. A potential explanation for the increase in nanosphere size is that PEG-PEI copolymers, while introduced into the primary aqueous phase, are also soluble in the organic phase and thus can become incorporated within it, or on the surface of the nanospheres. The amine groups of surface bound PEG-PEI copolymers can interact with carboxylic acid groups on the nanosphere surface, and may act as an electrostatic crosslinker between nanospheres that induces particle aggregation.

In an effort to obtain smaller mean particle sizes, studies were carried out to examine the influence of PVA concentration, sonication intensity, and sonication volume on the size of unloaded PLGA nanospheres. Pilot studies showed that at a given sonication intensity, the mean nanosphere size was decreased up to 30 nm by increasing the concentration of PVA (w/v) from 2 to 10% (data not shown), an observation that has been seen in previous studies. However, mass yield decreased by nearly 20% at both 8 and 10% PVA concentrations, negating any small advantage that would be obtained by the decrease in size. Thus, 5% PVA concentration was chosen for all subsequent experiments.

**Figure 2:**
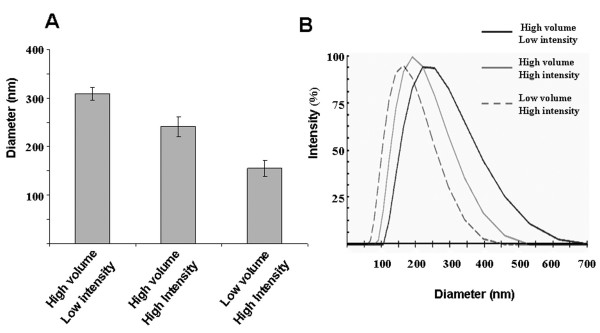
**Influence of sonication intensity and vessel volume on the mean size and size distribution of unloaded 72 kDa lauryl ester end-capped PLGA nanospheres**. (**A**) Mean diameter determined by DLS is shown for the nanospheres prepared in either a high or low volume chamber and with either low intensity (38 W) or high intensity (52 W) sonication. Three separate samples were evaluated for each group. **(B) **Size distribution based on the DLS measurements is shown for a single sample from each of the groups in panel A. Smaller and more uniform nanospheres were obtained using high sonication intensity in a low volume chamber.

**Figure 3:**
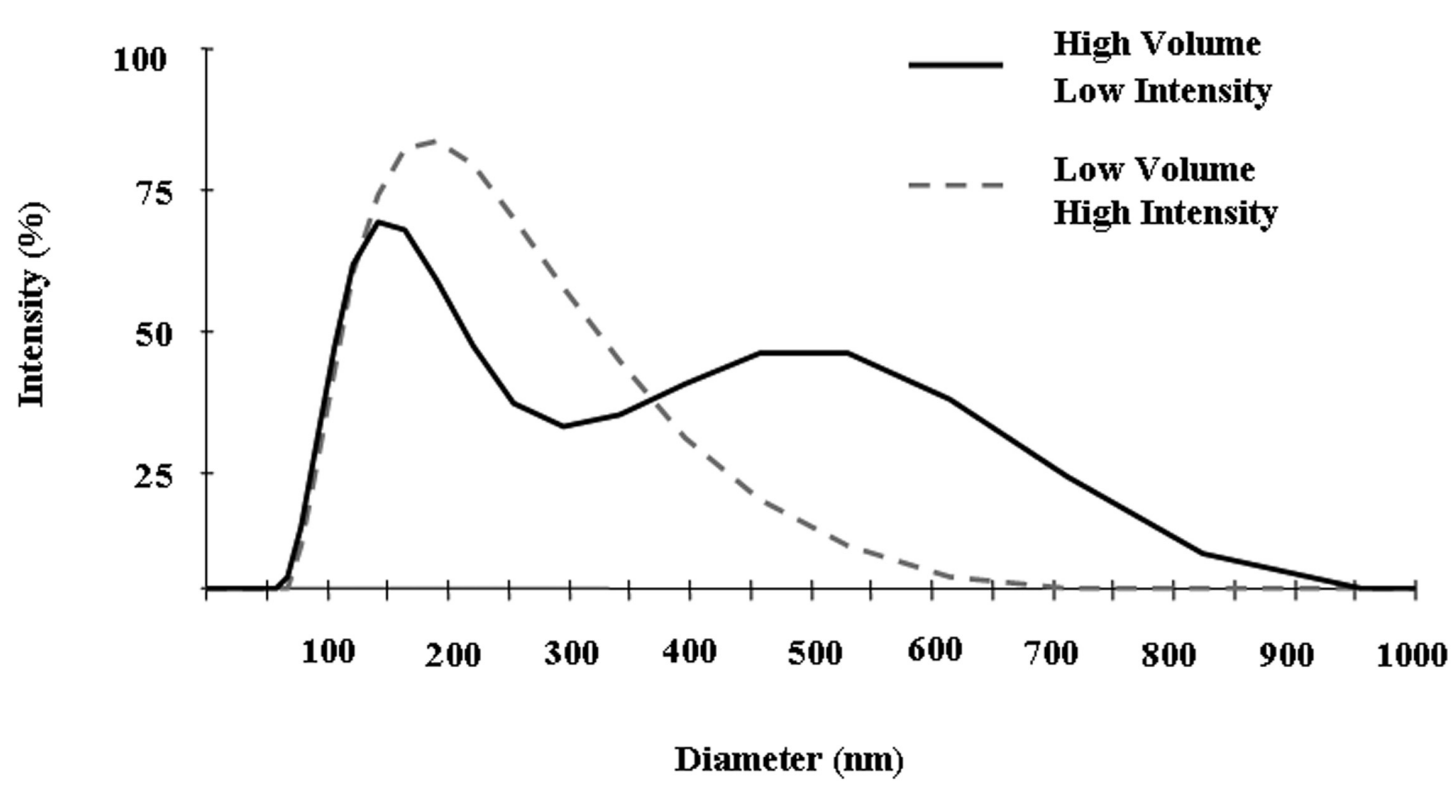
**Influence of sonication intensity and vessel volume on the size of 72 kDa lauryl ester end-capped nanospheres encapsulated with PEG-PEI-AO polyplex**. The size distribution within a single prepared sample of nanospheres formulated in a high volume chamber with a low intensity (38 W) sonication (full line) and a low volume chamber with a high intensity (51 W) sonication (broken line) were determined using DLS. Polyplex loaded nanospheres formulated with a low volume chamber and a high sonication intensity show a more uniform size distribution and smaller mean size.

Next, we compared the influence of sonication energy on size distribution of unloaded PLGA nanospheres. We found that mean size was reduced by increasing sonication energy from 38 W to a maximum of 51 W, producing nanospheres with mean sizes of 309.7 ± 13.2 nm and 241.8 ± 20.2 nm, respectively (Figure 2A). We further reasoned that sonication in smaller vessels may enhance the energy dissipation into the system. Thus, we also compared the size distribution of nanospheres formed by sonication in low and high volume chambers. We found that splitting the secondary emulsion into three 20 ml glass scintillation vials containing about 8.3 mL of solution each, sonicating at 52 W, and recombining the solutions, produced nanospheres with markedly reduced diameters (154.8 ± 16.4 nm). The size distributions of the samples are demonstrated to be more uniform with increased sonication intensity and lower volume (Figure 2B), indicated by the decreased half peak widths (calculated as 144 nm, 107 nm, and 95 nm for 38 W high volume sonication, 52 W high volume sonication, and 52 W low volume sonication, respectively). Importantly, this improved protocol also facilitated formation of smaller (206.9 ± 42.9 nm) and more uniform PLGA nanospheres when loaded with PEG-PEI-AO polyplex compared to the original protocol (Figure 3). Therefore, the lower volume chamber and higher intensity sonication were used for all subsequent experiments.

**Figure 4:**
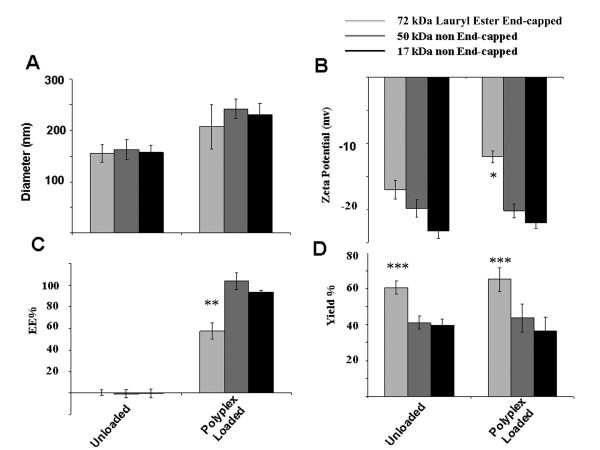
**Effect of PLGA polymer composition on the properties of resultant nanospheres**. PLGA (50:50) at molecular weights of 72 kDa, 50 kDa, and 17 kDa was used to encapsulate AO. The 50 kDa and 17 kDa polymers did not have lauryl ester end groups but unmodified carboxylic acid end groups instead. Measurements were done on unloaded and PEG-PEI-AO polyplex loaded nanospheres. The following properties of the nanospheres were evaluated: **(A) **Mean diameters measured by DLS. No statistical difference was observed between the nanospheres formulated using the three different PLGA polymers for either loaded or unloaded nanospheres (P > 0.05). **(B) **Surface charge (evaluated by zeta potential analysis) was determined by light scattering. A significant difference in zeta potential was seen between nanospheres formulated using each of the three different PLGA polymers for both polyplex loaded and unloaded nanospheres (P < 0.05). A significantly less negative zeta potential was observed for polyplex loaded compared to unloaded nanopsheres formulated using 72 kDa endcapped PLGA (*P = 0.027). No difference in zeta potentials were seen between polyplex loaded and unloaded groups for 50 kDa and 17 kDa PLGA. **(C) **Encapsulation efficiency (EE) of unloaded nanospheres and PEG-PEI-AO loaded nanospheres. Non-endcapped 50 kDa and 17 kDa PLGA polymers showed significantly higher encapsulation efficiencies compared to endcapped 72 kDa PLGA (**P < 0.05). **(D) **Polymer yield for the unloaded nanospheres and PEG-PEI-AO loaded nanospheres. The yield for 72 kDa PLGA was moderately higher than for 50 kDa and 17 kDa PLGA polymers for both unloaded and loaded samples (***P < 0.05). All measurements were repeated in triplicate from independently prepared samples.

The next step was to evaluate the influence of the PLGA composition on the nanocapsule properties. We selected lower molecular weight non-endcapped PLGA compositions (50 kDa and 17 kDa) that we expected to have faster degradation rates. Unloaded nanospheres showed mean sizes of 154.8 ± 16.4 nm, 162.5 ± 19.5 nm, and 160.1 ± 16.6 nm, for the 72 kDa, 50 kDa, and 17 kDa PLGA polymers, respectively (Figure 4A). Nanospheres loaded with PEG-PEI-AO showed mean sizes of 206.9 ± 42.9 nm, 241.7 ± 18.7 nm, and 231.0 ± 21.4 nm, for 72 kDa, 50 kDa, and 17 kDa PLGA, respectively (Figure 4A). Overall, whether loaded or unloaded, there were no significant differences in nanosphere size between the three different MW PLGA polymers that were investigated (P > 0.05). However, PLGA nanospheres loaded with PEG-PEI-AO were about 33–44% larger than un-loaded PLGA.

The surface charge of the PLGA nanospheres was evaluated by measuring zeta potential in dilute PBS. As expected, unloaded nanospheres formulated using lauryl ester end-capped 72 kDa PLGA showed the lowest zeta potential (-17.0 ± 1.4 mV) compared to non end-capped 50 kDa PLGA (-19.8 ± 1.2 mV) and non end-capped 17 kDa PLGA (-24.4 ± 1.1 mV) (Figure 4B), with significant differences between all three groups (P < 0.05). Although statistically significant, the influence of ester end-capping on zeta potential was surprisingly small. This may be due to hydrolysis of the PLGA chains during the solvent evaporation step, causing exposure of more carboxylic acid end groups.

The zeta potential of 50 kDa and 72 kDa PLGA nanospheres that were encapsulated with PEG-PEI-AO polyplexes was -20.1 ± 1.0 mV and -22.8 ± 1.8 mV, respectively, which was not significantly different than the respective un-encapsulated nanospheres (P > 0.05). However, the loading of PEG-PEI-AO polyplexes into endcapped 72 kDA PLGA nanospheres resulted in a marked neutralization of the zeta potential (-12.0 ± 0.8 mV; Figure 4B). The amelioration of the surface charge may be due to excess un-encapsulated cationic PEG-PEI-AO adsorbed to the surface of the nanospheres during the formulation procedure. Non-endcapped PLGA polymers appear to encapsulate polyplexes within the polymer matrix with a much greater efficiency than ester end-capped PLGA (see below), which may eliminate surface adsorption of PEG-PEI-AO.

As shown in Figure 4C the EE for 72 kDa PLGA was only about 57.6 ± 7.6%, which was significantly less than for both 50 kDa PLGA (103.8 ± 7.8%) and 17 kDa PLGA (93.3 ± 1.5%) (P < 0.01). On the other hand, the nanosphere yield for the 72 kDa PLGA preparation was moderately, but significantly, greater than for the 50 kDa PLGA and 17 kDa PLGA (P < 0.05) (Figure 4D). The higher level of entrapment is most likely attributed to interaction between carboxylic acid end groups on the PLGA interacting with amine groups on the PEG-PEI. This idea is supported by De Rosa et al [39,49] who showed significantly higher EE of oligonucleotides within PLGA microspheres in the presence of PEI. The increased level of AO entrapment in the nanospheres highlights another advantage of using PEG-PEI in PLGA formulations. Due to the low AO encapsulation efficiency within nanospheres formulated using lauryl ester end-capped PLGA, the following release kinetic studies and *in vivo *evaluation of the PEG-PEI-AO nanospheres focused on formulations using non-endcapped PLGA.

**Figure 5:**
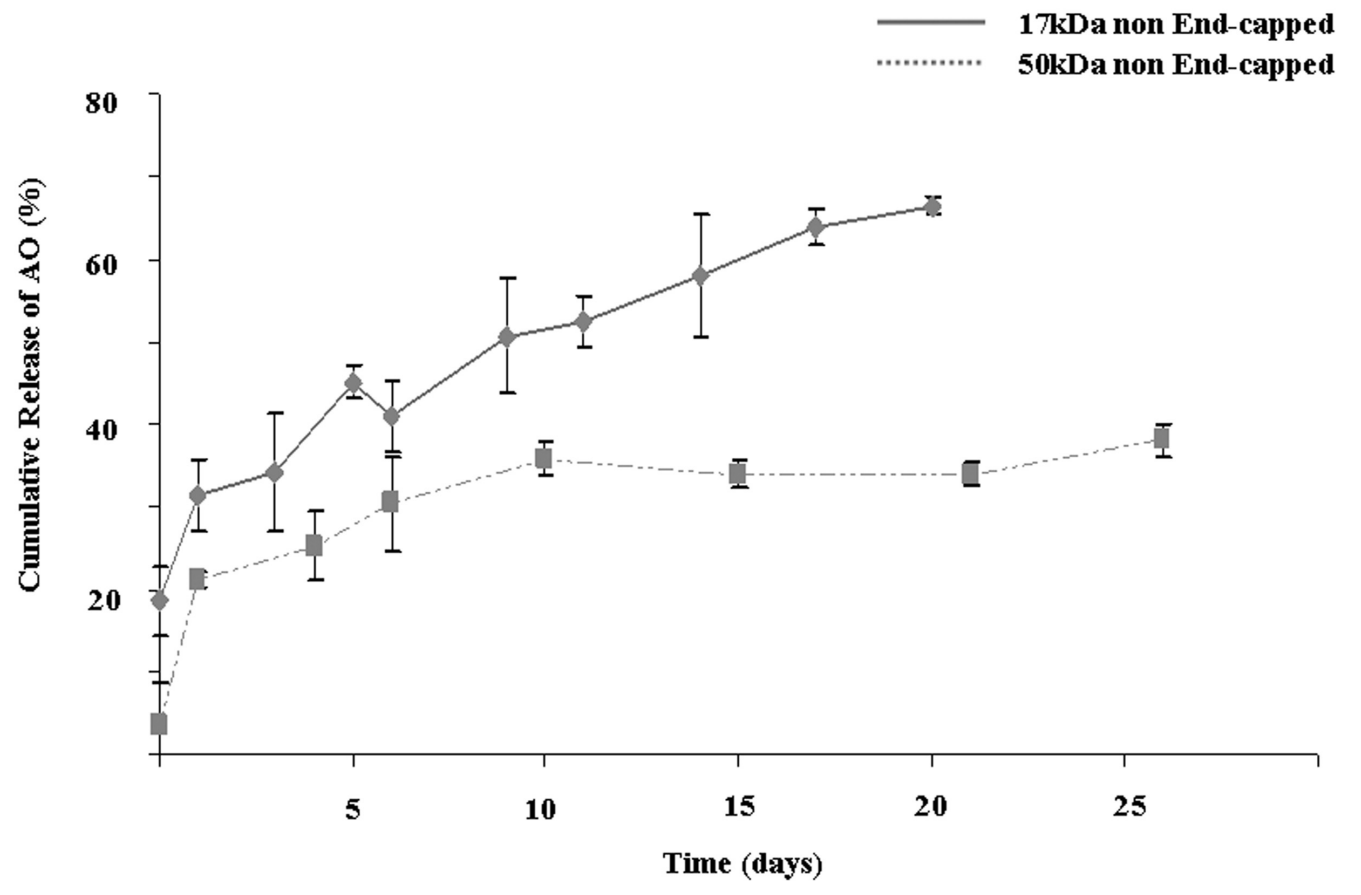
**Release kinetics of PEG-PEI-AO polyplex from PLGA nanospheres**. Nanospheres were formulated with PLGA polymers with molecular weights of 50 kDa (full line), and 17 kDa (broken line). Measurements were repeated in triplicate for each group.

The release rate of PEG-PEI-AO from the PLGA nanospheres was measured over the course of 26 days. The rate of AO release increased with decreasing PLGA molecular weight, giving a cumulative release of only 38.0 ± 2.0% for 50 kDa PLGA, but significantly higher cumulative release of 66.5 ± 1.0% for the 17 kDa PLGA (P < 0.01) (Figure 5). The burst release of PEG-PEI-AO from the 17 kDa PLGA over the first 24 hours was nearly 20% of the total payload, a rate approximately 4 times greater than for the 50 kDa PLGA (5%). Interestingly, pilot experiments showed almost no release of polyplexes from nanospheres formulated with 72 kDa PLGA (data not shown). The reason for this is not clear, but we presume that the end-capping and high molecular weight of the PLGA significantly retarded the rate of hydrolysis, and therefore AO release.

To assess functionality of the PLGA compounds we utilized a well characterized paradigm of AO-mediated dystrophin expression after intramuscular injections in dystrophic *mdx *mice. The *mdx *mouse contains a stop codon in exon 23 of the dystrophin gene, which results in production of truncated and non-functional dystrophin, resulting in a dystrophic phenotype that resembles DMD. We and others have recently used an AO that causes skipping of dystrophin exon 23 to rescue dystrophin expression in skeletal muscle fibers of *mdx *mice after intramuscular injections [8,24,25,50-52]. Because the release of PEG-PEI-AO from the 17 kDa PLGA was more rapid and complete than other PLGAs, we utilized this formulation exclusively in the functional studies.

**Figure 6:**
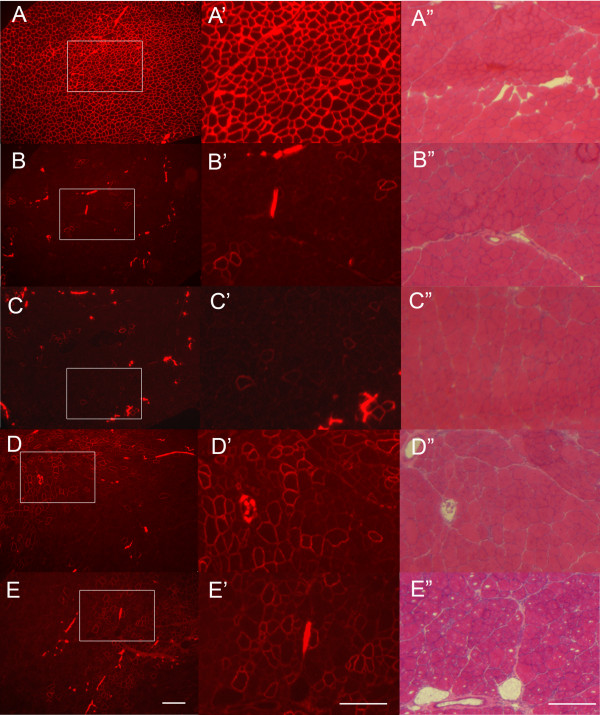
**Dystrophin induction in TA muscles of *mdx *mice 3 weeks after intramuscular injections of AO uwith and without polymer carriers**. Muscles were injected on days 0, 3, and 6 and harvested at 3 weeks after the initial injection. Dystrophin immunolabeling of TA muscle cross-sections at two different magnifications and H&E staining of serial sections are shown for (a-a") normal, (b-b") *mdx *untreated, (c-c") *mdx *injected with AO alone, (d-d") *mdx *injected with PEG-PEI-AO polyplex, and (e-e") *mdx *injected with PLGA (17 kDa) nanospheres encapsulated with the PEG-PEI-AO polyplex.

**Figure 7:**
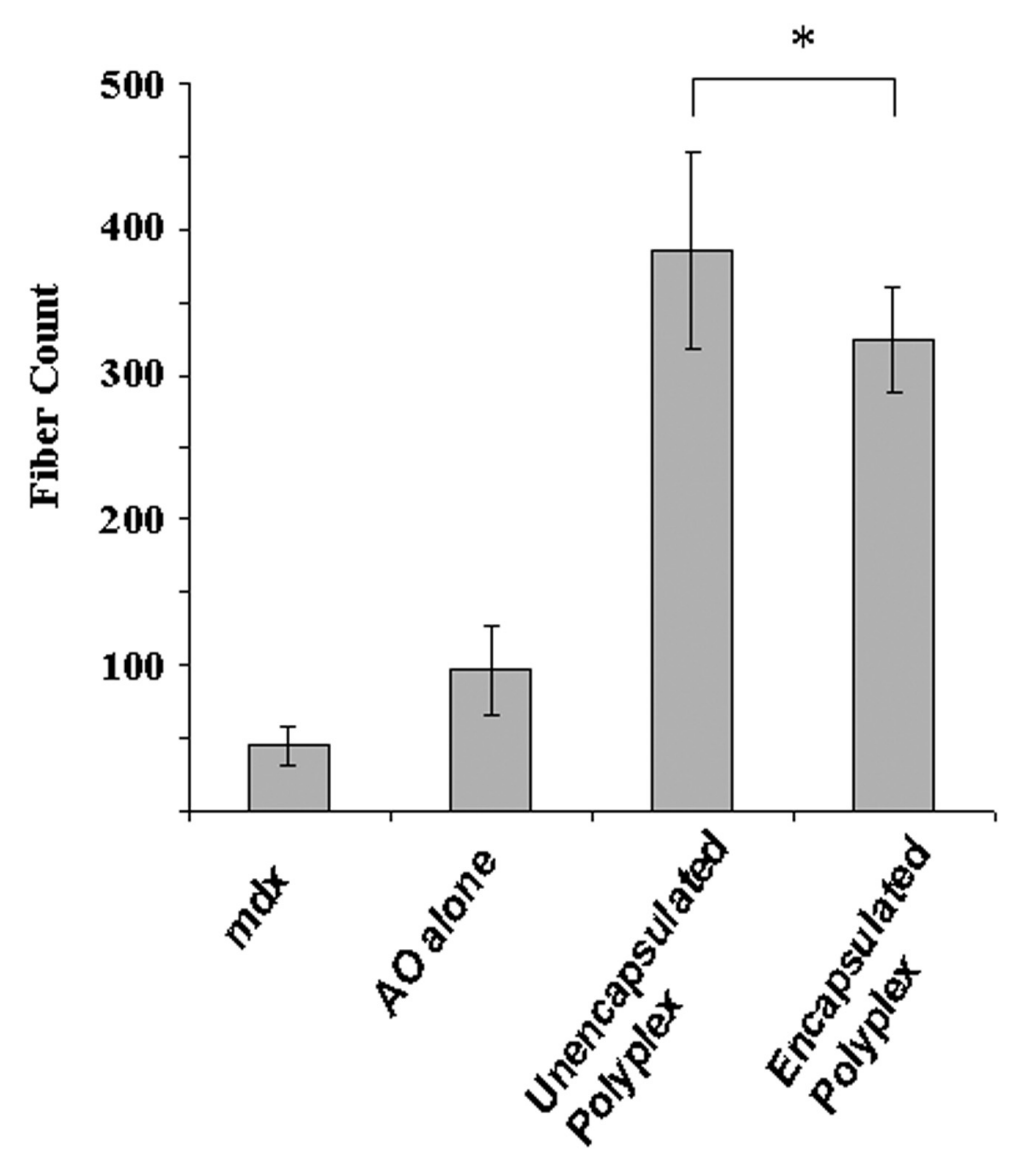
**Comparison of the number of dystrophin-positive fibers in TA muscles of *mdx *mice at 3 weeks after 3 intramuscular injections of PEG-PEI-AO polyplex and PLGA (17 kDa) nanospheres encapsulated with the PEG-PEI-AO polyplex**. Results are also shown for injections of AO alone and untreated *mdx *muscles which are known to contain a small number of revertant fibers. All treated muscles were given 5 μg AO per injection. Both PLGA encapsulated polyplex and unencapsulated polyplex showed a significantly higher number of dystrophin positive fibers compared to AO alone injected or untreated *mdx *muscle (*P < 0.05). No statistical difference in dystrophin positive fibers was observed between untreated muscle and AO alone injected groups. Fiber counts were determined from four independently treated muscles in each group (N = 4).

**Figure 8:**
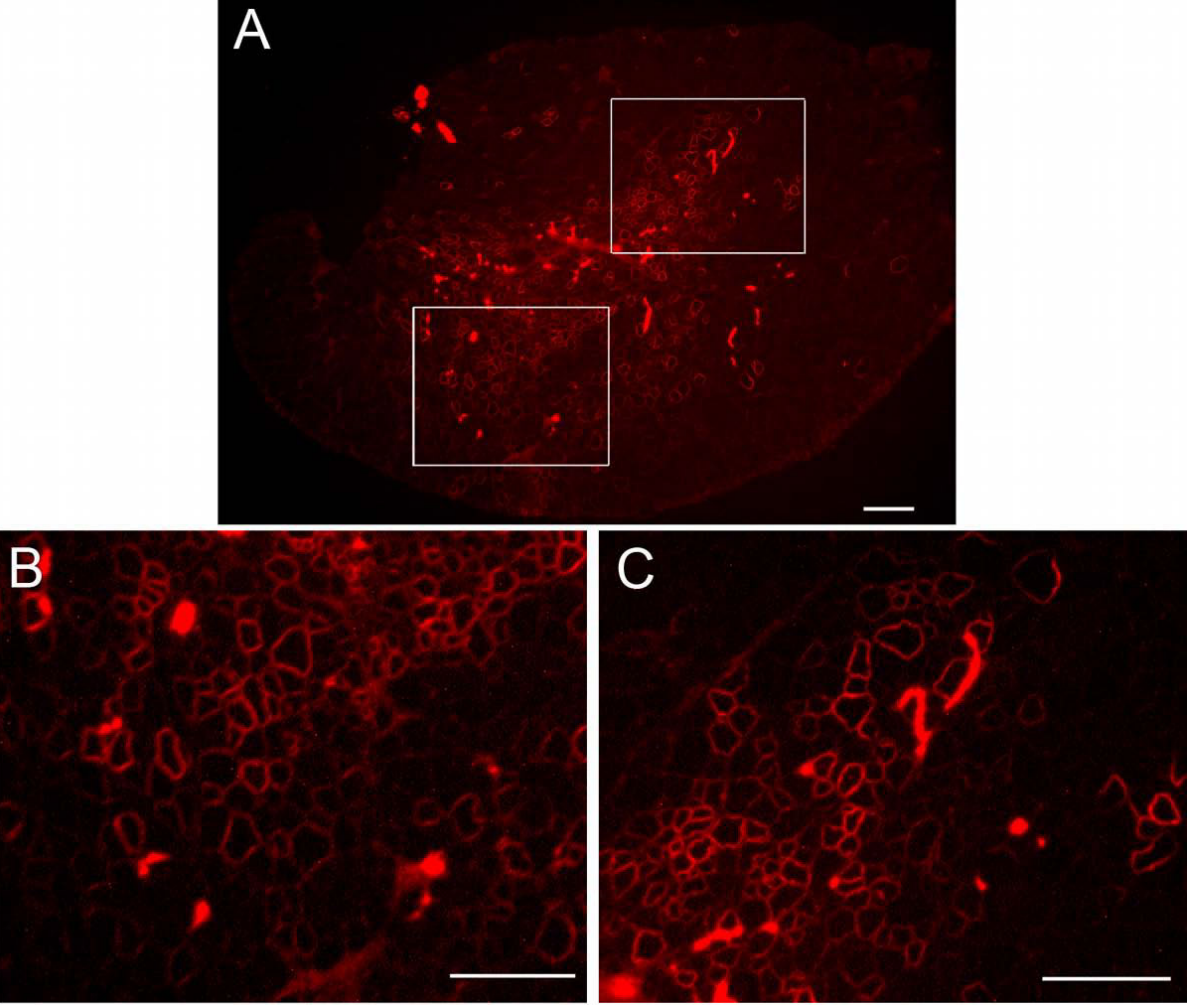
**Dystrophin expression in a whole transverse section from TA muscle of an *mdx *mouse after three intramuscular injections of PEG-PEI-AO encapsulated in PLGA (17 kDa) nanospheres**. Dystrophin immunolabeling is shown for the whole transverse section (A) and the two boxed regions are shown at higher magnification (B, C).

PLGA nanospheres (17 kDa) loaded with PEG-PEI-AO were injected into TA muscles of *mdx *mice on day 0, 3 and 6 using 5 μg of AO per injection, and muscles were harvested 3 weeks after the first injection. Immunolabeling of transverse sections obtained from the mid-portion of TA muscles revealed that intramuscular injections of PLGA-PEG-PEI-AO resulted in the appearance of focal regions containing densely distributed dystrophin-positive fibers (Figure 6). On average, muscles treated with PLGA-PEG-PEI-AO contained significantly more dystrophin-positive fibers (324.8 ± 71.6) compared to muscles injected with AO alone (96.4 ± 62.6) or control uninjected *mdx *muscles (45 ± 26.9; P < 0.01; Figure 7). Immunolabeling of entire transverse sections demonstrates that although there were pockets of densely populated dystrophin-positive fibers, over 50% of the muscle cross-sections remained devoid of those fibers, owing to the incomplete diffusion of the PLGA nanocapsules throughout the muscle (Figure 8). Together, these data illustrate that PEG-PEI-AO is released from PLGA nanospheres in vivo and that the AO is able to maintain functionality, as indicated by induction of dystrophin expression.

Intramuscular injections of un-encapsulated PEG-PEI-AO polyplexes also resulted in improved expression of dystrophin-positive fibers (Figure 6), with no significant difference in the number of dystrophin-positive fibers between muscles injected with PLGA-PEG-PEI-AO or un-encapsulated PEG-PEI-AO (P = 0.49; Figure 7). These results show that this particular formulation of PEG-PEI copolymer, comprised of high MW PEI (25 kDa) and long PEG chains (5 kDa) appears to function as a fairly efficient carrier on its own for delivery of AO to myofibers.

Although not systematically studied, western blots indicated that both PEG-PEI-AO and PLGA-PEG-PEI-AO produced only about 5–10% of normal levels of dystrophin expression (data not shown). This was much less than the 20–30% of dystrophin expression we obtained with PEG-PEI copolymers comprised of low MW PEI (2 kDa) [24,25], but was still much better than the dystrophin expression found with AO alone (~0–2% of normal). Further studies must be done to evaluate whether encapsulation of PEG-PEI-AO containing low MW PEI2K in PLGA will perform better than the high MW PEI25K. As discussed above, the release rate from the 17 kDa PLGA nanospheres was quite slow, reaching only 60% release after 3 weeks. We expect that the advantage of encapsulating PEG-PEI-AO in PLGA may become more significant when the functionality (dystrophin expression) is measured over a longer time range. Histological analysis of muscle morphology did not reveal any overt signs of cytotoxicity at 3 weeks after injection, indicating that the degradable PLGA nanospheres may be suitable for longer term application to muscles (Figure 6).

## Conclusion

In this study, formulation conditions were established for encapsulating PEG-PEI-AO polyplexes within biodegradable PLGA nanospheres. Although several previous studies reported encapsulation of cationic polymers complexed to nucleic acids within PLGA structures, these studies focused on relatively large micron-sized PLGA formulations, that limits their usefulness for some *in vivo *applications (reviewed in Capan et al. [53]). In the present study, formulation parameters were chosen to allow for nearly 100% encapsulation efficiency of positively-charged PEG-PEI-AO polyplexes within PLGA nanospheres (200–300 nm) that shielded the surface charge of the cationic polyplexes and showed a surprisingly uniform size distribution. The PLGA nanospheres exhibited sustained release of the PEG-PEI-AO polyplexes in solution. Immunohistochemical analysis demonstrated AO-mediated dystrophin expression 3 weeks after intramuscular injections in *mdx *mice of the PLGA-encapsulated PEG-PEI-AO polyplexes. A drawback to the study is that the dystrophin expression levels were not improved using encapsulated polyplexes compared to unencapsulated polyplexes. One reason for this may be incomplete release of AO at 3 weeks following injection. This study demonstrated the feasibility of PEG-PEI-AO encapsulation, however further studies evaluating dystrophin expression at multiple time points are warranted to fully realize their potential *in vivo*. To our knowledge, the present study is the first to demonstrate the feasibility of internalizing PEG-PEI-AO polyplexes within PLGA nanospheres for *in vivo *applications. The nanometer size, charge shielding, and controlled release properties of the PLGA carriers should offer significant improvement in the biodistribution and sustained delivery of AO complexed with the PEG-PEI copolymers, enhancing the utility of this popular carrier for *in vivo *usage.

## Competing interests

The authors declare that they have no competing interests.

## Authors' contributions

SS designed and carried out PLGA formulation and characterization studies, data analysis and statiscal analysis, and drafting of the manuscript. RS carried out animal injections and immunohistochemistry for the *in vivo *testing of the polyplex loaded PLGA nanospheres. MW and GL were involved with the design, coordination, data analysis, and drafting of the manuscript.
